# Translational evidence for RRM2 as a prognostic biomarker and therapeutic target in Ewing sarcoma

**DOI:** 10.1186/s12943-021-01393-9

**Published:** 2021-07-27

**Authors:** Shunya Ohmura, Aruna Marchetto, Martin F. Orth, Jing Li, Susanne Jabar, Andreas Ranft, Endrit Vinca, Katharina Ceranski, Martha J. Carreño-Gonzalez, Laura Romero-Pérez, Fabienne S. Wehweck, Julian Musa, Felix Bestvater, Maximilian M. L. Knott, Tilman L. B. Hölting, Wolfgang Hartmann, Uta Dirksen, Thomas Kirchner, Florencia Cidre-Aranaz, Thomas G. P. Grünewald

**Affiliations:** 1 Hopp Children’s Cancer Center Heidelberg (KiTZ), Heidelberg, Germany; 2 Division of Translational Pediatric Sarcoma Research (B410), German Cancer Research Center (DKFZ) & Hopp-Children’s Cancer Center (KiTZ), Im Neuenheimer Feld 280, 69210 Heidelberg, Germany; 3 Max-Eder Research Group for Pediatric Sarcoma Biology, Institute of Pathology, Faculty of Medicine, LMU Munich, Munich, Germany; 4 Pediatrics III, West German Cancer Centre, University Hospital Essen, Essen, Germany; 5 German Cancer Consortium (DKTK), partner site Essen, Essen, Germany; 6 Department of General, Visceral and Transplantation Surgery, Heidelberg University Hospital, Heidelberg, Germany; 7 Light Microscopy Facility, German Cancer Research Center (DKFZ), German Cancer Consortium (DKTK), Heidelberg, Germany; 8 Division of Translational Pathology, Gerhard-Domagk-Institute for Pathology, University Hospital Münster, Münster, Germany; 9 Institute of Pathology, Faculty of Medicine, LMU Munich, Munich, Germany; 10 German Cancer Consortium (DKTK), partner site Munich, Munich, Germany; 11 Institute of Pathology, Heidelberg University Hospital, Heidelberg, Germany

**Keywords:** Ewing sarcoma, RRM2, Targeted therapy, Prognostic biomarker, Paediatric oncology, Triapine, Chemoresistance

## Main text

Ewing sarcoma (EwS) is an aggressive bone- or soft tissue-associated malignancy, characterised by the fusion oncoprotein EWSR1-FLI1 [1]. Over the past decades further therapeutic development for this devastating childhood tumour has remained relatively stagnant [2], especially for patients with metastatic or recurrent disease [3, 4]. To develop more effective and specific treatment options we investigated potential therapeutic targets by exploring putative downstream genes of EWSR1-FLI1.

**Fig. 1:**
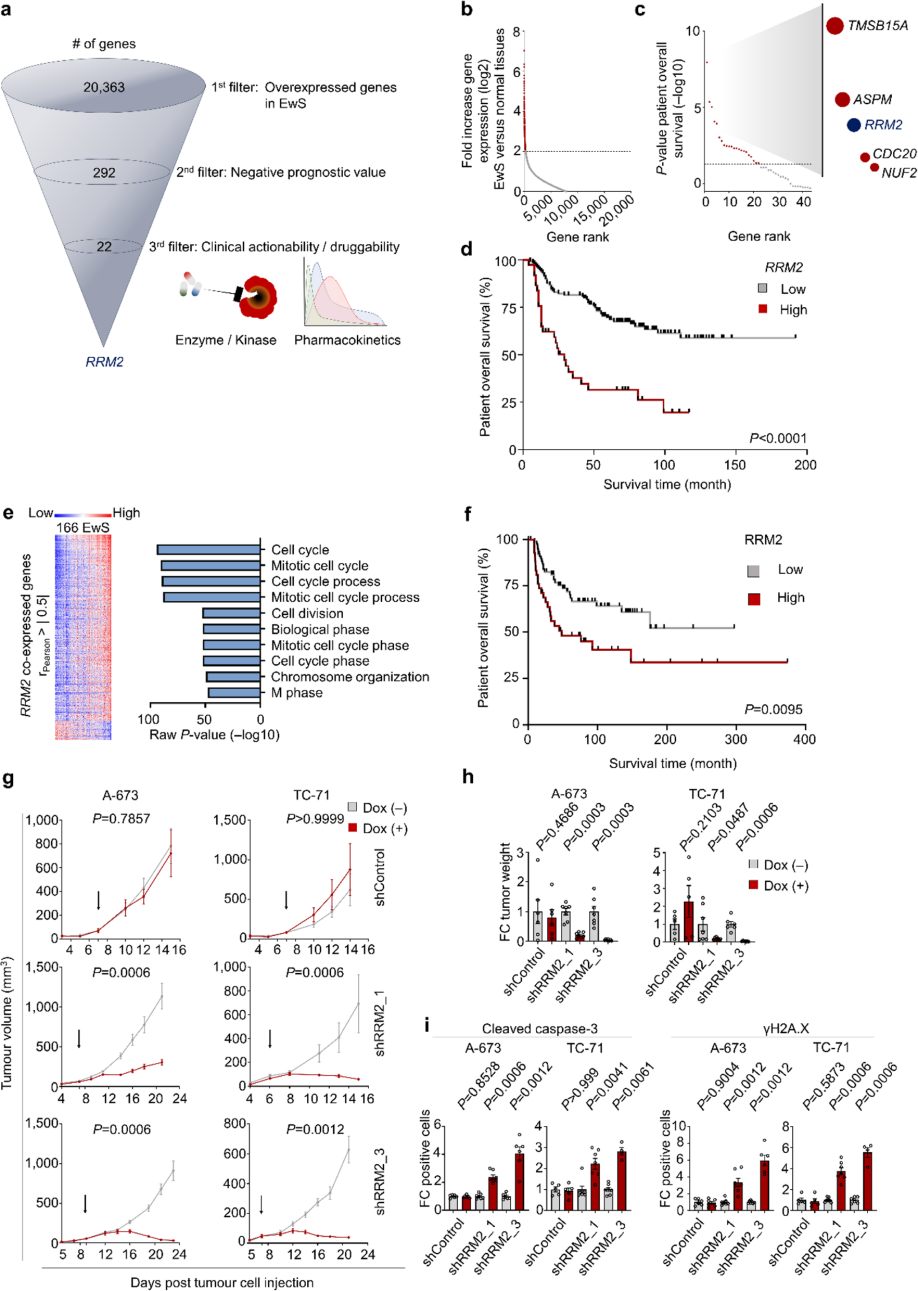
*RRM2* is highly overexpressed in EwS, correlates with poor patient outcome, and constitutes a putative therapeutic target. **a** Schematic description of the filtering process for identification of therapeutically relevant target candidates. **b** Analysis of *RRM2* mRNA expression levels in 50 EwS primary tumours compared to 929 normal tissues samples from 71 tissue types. Data are shown as log2 fold increase normalized to expression values of normal tissues. The dotted line indicates the cut-off value of 2 for candidate selection. **c** Analysis of overall survival time of 166 EwS patients stratified for candidate gene expression. *P*-values (–log10) were determined in Kaplan–Meier analyses using a Mantel–Haenszel test (Bonferroni-adjusted for multiple testing). The dotted line indicates a significance value of 1.3. **d** Kaplan–Meier survival analysis of 166 EwS patients stratified by the 78^th^ percentile *RRM2* expression. *P*-value determined by log-rank test. **e** Left: Heat map for gene expression which is positively or negatively correlated with *RRM2* expression in 166 EwS. Right: Gene ontology (GO) enrichment analysis of *RRM2* and its co-expressed genes derived from gene expression data sets of 166 EwS tumours. Pearson correlation coefficients between *RRM2* and other genes were determined, of which those with |r_Pearson_|> 0.5 were further analysed by GO enrichment analysis. **f** Kaplan–Meier survival analysis of 122 EwS patients stratified by RRM2 protein expression (low IRS ≤ 2, high IRS > 2). *P*-values were determined by log-rank test. **g** Analysis of tumour growth of EwS cell lines A-673 and TC-71 harbouring Dox-inducible shRRM2 constructs or non-targeting shRNA (shControl) xenografted in NSG mice. Once tumours were palpable, animals were randomized in Dox ( +) or Dox (–) group. Tumour growth on time course and **h** Tumour weight at the experimental endpoint. Arrows indicate treatment start. Values were normalized to Dox (–). Horizontal bars represent means and whiskers SEM. FC, fold change. *P*-values were calculated at the experimental endpoint with two-sided (tumour growth) or one-sided (tumour weight) Mann–Whitney test. **i** Quantification of positive cells for cleaved caspase-3 (CC3) (left) and γH2A.X (right). Values were normalized to Dox (–). Horizontal bars represent means and whiskers SEM. FC, fold change. *P*-values were calculated at the experimental endpoint using a two-sided Mann–Whitney test

We took advantage of publicly available ‘omics’ data and filtered them in a multi-step approach (Fig. 1a): First, we interrogated a gene expression dataset comprising 50 primary EwS and 929 samples from 71 normal tissue types to identify overexpressed genes (min. log2 fold increase = 2) in EwS, which yielded 292 candidates (Fig. 1b, Supplementary Table 1). Second, we filtered for those genes whose overexpression was significantly negatively correlated with patients’ overall survival in a dataset of matched gene expression and survival data of 166 EwS patients [5] that covered 280 of the 292 overexpressed genes (96%) (Fig. 1c), identifying 22 candidates (Supplementary Table 1). Third, we focused on druggable targets possessing kinase or other enzymatic functions for which specific inhibitors and their pharmacokinetic data were available, but were still not (pre)clinically tested in EwS. This survey identified ribonucleotide reductase regulatory subunit M2 (*RRM2*) as the single putative target with a prominently negative association with patients’ overall survival (Fig. 1d). The ribonucleotide reductase (RNR) catalyses the conversion of ribonucleoside diphosphates to deoxyribonucleoside diphosphates, the rate-limiting process for de novo deoxyribonucleoside triphosphates synthesis. RNR is composed of two subunits, ribonucleotide reductase catalytic subunit M1 (RRM1) and either RRM2 or ribonucleotide reductase regulatory *TP53* inducible subunit M2 (RRM2B) [6]. Notably, *RRM2B* is neither overexpressed in EwS nor negatively correlates with patients’ outcome (Supplementary Figs. 1a,b), and *RRM1* is far less overexpressed in EwS compared to *RRM2* (Supplementary Figs. 1a,b,c). Similar to primary EwS tumours, assessment of transcriptomes from 18 EwS cell line models (including A-673 and TC-71) also exhibited that, while *RRM2* and *RRM1* were similarly highly expressed in EwS cell line models, *RRM2* was on average ~ ninefold higher expressed than *RRM2B* (*P* < 0.0001). These observations, together with the absence of a negative survival association of *RRM2B* in EwS (Supplementary Fig. 1b), suggested that RRM2B, although being structurally similar to RRM2 [6], may play a subordinate role in EwS.

Prior reports suggested that RRM2 may contribute to the proliferative phenotype of EwS [7, 8]. However, its role in primary EwS tumours remains unclear. To gain first insights into the biological function of *RRM2* in EwS, we carried out gene ontology (GO) enrichment analysis of *RRM2* co-expressed genes in 166 EwS tumours, which revealed that high *RRM2* expression is closely correlated with cell proliferation-associated gene signatures (Fig. 1e), suggesting that high *RRM2* expression may contribute to an aggressive clinical course by promoting tumour growth. Next, we analysed the potential association between RRM2 protein levels, clinicopathological prognostic factors, and clinical outcomes in tissue microarrays (TMA) from EwS tumours of 122 patients (Supplementary Table 2, Supplementary Fig. 2a). In agreement with the findings at the mRNA level (Fig. 1d), high RRM2 protein expression was significantly (*P* = 0.0095) associated with poor overall survival (Fig. 1f). Correspondence analyses of individual cohorts and the joint-cohort (after exclusion of 6 samples (3.6%) from the mRNA-cohort that were in overlap with the TMA cohort) revealed that high RRM2 expression was significantly associated with metastatic disease at diagnosis (*P* = 0.0004) and occurrence of metastatic and/or local relapse (*P* = 0.0095; only available for the TMA cohort) (Supplementary Table 3), supporting that high RRM2 expression promotes an aggressive phenotype. Conversely, *RRM2* inhibition by doxycycline (Dox)-inducible shRNA-mediated gene silencing inhibited proliferation and clonogenic growth of three EwS cell lines, and induced cell death in vitro (Supplementary Figs. 2b,c). Consistent with these functional experiments, transcriptome profiling upon *RRM2* silencing in two EwS cells demonstrated downregulation of cell cycle and proliferation-associated gene signatures (Supplementary Fig. 2d). Similarly, *RRM2* knockdown significantly reduced tumour growth of two xenografted EwS cells (Figs. 1g,h). This antineoplastic effect was accompanied by increased apoptosis and DNA damage, as assessed by immunohistochemistry for cleaved caspase 3 (CC3) and γH2A.X, respectively (Fig. 1i, Supplementary Fig. 2e).

**Fig. 2:**
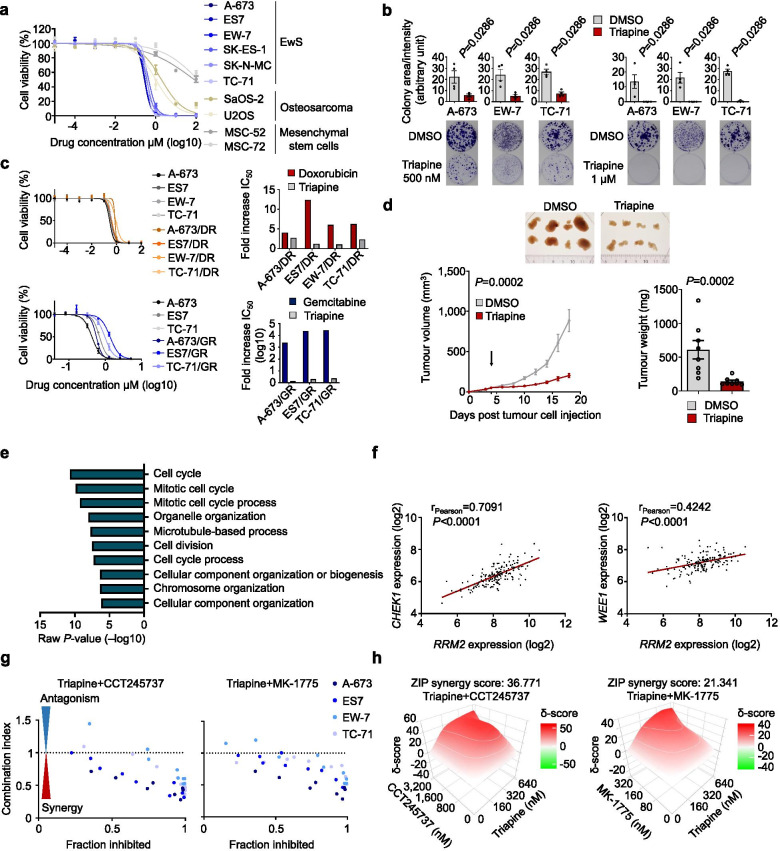
Triapine inhibits EwS growth in vitro and in vivo and synergise with cell cycle checkpoint inhibitors in vitro. **a** Dose–response analysis of triapine in EwS, osteosarcoma and mesenchymal stem cells. **b** Analysis of clonogenic growth of A-673, TC-71, and EW-7 EwS cells upon triapine treatment. **c** Left: Dose–response analysis of triapine in chemoresistant EwS cells (A-673/DR or A-673/GR). Right: magnitudes of doxorubicin or gemcitabine resistance shown by fold increase in IC_50_ compared to those of parental cells. **d** Analysis of tumour growth upon triapine treatment in A-673 cell line in vivo. Images of resected xenografts (upper), tumour growth (lower left), and tumour weight at the experimental endpoint (lower right) of NSG mice xenografted with A-673 EwS cells upon treatment with triapine. Once tumours reached 5 mm in average diameter, animals were randomized in treatment group (30 mg/kg i.p.) or control group (DMSO) (*n* = 8 animals per group). The arrow indicates treatment start. Horizontal bars represent means and whiskers SEM. *P*-values were calculated at the experimental endpoint with two-sided (tumour growth) or one-sided (tumour weight) Mann–Whitney test. **e** Integrative Gene Ontology (GO) enrichment analysis of gene expression microarray data generated in A-673 and ES7 cells after *RRM2* silencing or pharmacological RRM2 inhibition by triapine (corresponding IC_50_ of 0.44 µM or 0.65 µM, respectively). **f** Correlation of gene expression between *RRM2* and *CHEK1* or *WEE1* in 166 EwS. Each dot represents an individual expression value. Solid red lines indicate a trend line created by a simple linear regression. *P*-values were calculated by a two-tailed t-test. **g** Drug interaction and combination efficiency analysis between triapine and CHEK1 inhibitor (CCT245737) or WEE1 inhibitor (MK-1775) in 4 EwS cell lines (A-673, ES7, EW-7, TC-71) assessed by combination index. CI value < 1 indicative of synergistic, CI = 1 additive, and CI > 1 antagonistic **h** Drug interaction and combination efficiency estimation between triapine and CHEK1 inhibitor (CCT245737) or WEE1 inhibitor (MK-1775) in A-673 EwS cell line assessed by SynergyFinder 2.0. ZIP synergy score > 10, likely to be synergistic; between –10 and 10, likely to be additive; < –10, likely to be antagonistic

Generally, the activity of RNR can be blocked by irreversible RRM1 inhibition using gemcitabine, or by RRM2-specific inhibitors such as hydroxyurea or the more potent compound triapine (alias 3-AP) [6, 9]. Although gemcitabine is used for palliative treatment of EwS patients, EwS tumours rapidly develop a relative resistance [10]. Consistently, we found that long-term treatment of EwS cells with ascending doses of either doxorubicin (A-673, ES7, EW-7, TC-71), gemcitabine (A-673, ES7, TC-71) or triapine (A-673) led to acquisition of relative resistance phenotypes in vitro (Supplementary Fig. 3a), where we noted a relatively fast and strong increase of the relative resistance towards gemcitabine (> 2,000-fold increase in IC_50_ within ~ 6 weeks), compared doxorubicin (~ fourfold increase in ~ 28 weeks) and triapine (~ sevenfold increase in ~ 20 weeks) (Supplementary Fig. 3b), further suggesting that gemcitabine has limited potential for clinical treatment with curative intent. Thus, we focused on triapine for further functional analyses. First, to assess functional dependency of triapine on *RRM2* expression, we performed drug-response assays using triapine in EwS cells with/without *RRM2* silencing, which demonstrated that knockdown of *RRM2* led to a ~ twofold decrease of the IC50 for triapine in A-673 EwS cells, indicating that higher doses of the drug are required to fully block RRM2 activity in case of high RRM2 expression (Supplementary. Figure 3c). Such differential effect on sensitivity towards triapine was not observed in A-637 cells expressing a non-targeting control shRNA. Moreover, we observed an ~ twofold increase of *RRM2* expression in triapine-resistant A-673 (A-673/TR) compared to parental A-673 EwS cells, suggesting that *RRM2* upregulation can be a potential mechanism for acquiring triapine-resistance in A-673 EwS cells (Supplementary Fig. 3d). Dose–response assays revealed that EwS cells were very sensitive towards triapine compared to osteosarcoma cells and non-transformed EwS patient-derived mesenchymal stem cells (mean IC_50_ values 0.35, 1.63, 101.63 µM, respectively) (Fig. 2a). Likewise, triapine treatment significantly reduced clonogenic growth of EwS cells at clinically relevant doses [11, 12] (Fig. 2b). Interestingly, doxorubicin or gemcitabine resistant EwS cells (designated EwS/DR or EwS/GR, respectively) still retained triapine sensitivity (Fig. 2c), suggesting therapeutic potential of triapine for EwS refractory towards conventional chemotherapy. Strikingly, we could confirm a significant reduction of tumour growth by triapine treatment compared to controls in a subcutaneous xenograft model (Fig. 2d). Although triapine treatment was accompanied by weight loss (on average ~ 5% at the experimental endpoint) (Supplementary Fig. 3e), no morphological changes of inner organs were observed including the gastrointestinal tract as assessed by histological analysis (Supplementary Fig. 3f). Although it is interesting to note that EwS cells resistant to gemcitabine, which covalently binds and thus inactivates RRM1 [13], still retain sensitivity towards triapine (Fig. 2c), it should be noted that triapine may not be entirely specific for RRM2. Triapine presumably disrupts a tyrosyl free radical by labilising di-iron molecules on the small subunits of ribonucleotide reductase [6, 14]. This proposed mechanism of action for triapine can clinically manifest as a reversible adverse effect such as methemoglobinemia, which is probably caused by the iron chelating effect of triapine, interrupting recovery cycles from methaemogloblin to haemoglobin [15]. To mitigate this toxicity, the small molecule COH29 has been developed that, upon binding to RRM2 subunits, interferes with the molecular interface of RRM1 and RRM2 subunits and thus inhibits its reductase function [14]. Yet, its clinical efficacy and safety remain to be investigated. Another approach for more specific RRM2 inhibition has been undertaken with antisense oligonucleotide-based techniques, exemplified by therapeutic silencing of *RRM2* by GTI-2040, which, however, showed little clinical benefit in several clinical trials [16–18]. Hence, despite our data strongly support RRM2 as an actionable and valuable drug target in EwS, and triapine as a potential lead candidate drug for preferential RRM2 inhibition, the development of even more specific RRM2 inhibitors is desirable.

We next explored effective drug combinations with triapine. Based on known functions of RRM2 in DNA synthesis and DNA repair [6] we examined combinatory applications of triapine with standard chemotherapeutics, doxorubicin, etoposide or vincristine, or poly ADP-ribose polymerase (PARP) inhibitors. Unexpectedly, we observed rather antagonistic effects (Supplementary Figure 4a). To identify rational combinations, we analysed integrated transcriptome profiles of two EwS cells upon *RRM2* silencing and triapine treatment, revealing 263 commonly up- and downregulated genes (Supplementary Table 4). GO enrichment analysis demonstrated significant enrichment for cell cycle-associated processes, especially regulation of mitotic cell cycle-associated genes (Fig. 2e), which is consistent with the observation that RRM2 inhibition caused G1/S-phase cell cycle arrest [19, 20]. Thus, we reasoned that RRM2 may synergise with checkpoint inhibitors targeting CHEK1 (checkpoint kinase 1) or WEE1 (WEE1 G2 checkpoint kinase), which were highly significantly (*P* < 0.0001) co-expressed with *RRM2* in 166 EwS tumours (Fig. 2f). In drug combination assays we observed a strong synergism between triapine and a CHEK1 inhibitor (CCT245737) or a WEE1 inhibitor (MK-1775) across four EwS cells (Fig. 2g,h, Supplementary Fig. 4b). Overall, these results provide a rationale for therapeutic combination of triapine with cell cycle checkpoint inhibitors. A recent study pointed out that the drug combination of hydroxyurea and a CHEK1 inhibitor (GDC-0575) can circumvent toxicities caused by the combination of gemcitabine and GDC-0575, which may imply a more manageable combinatory application through RRM2 inhibition and CHEK1 inhibitors [21].

## Conclusions

Collectively, our results establish RRM2 as a promising actionable therapeutic target for EwS, even in chemotherapy-refractory cases, and suggest that the combination of triapine with cell cycle checkpoint inhibitors may be highly effective. Moreover, our integrative study of two independent cohorts provides evidence for RRM2 as novel and robust prognostic biomarker that can be readily assessed by immunohistochemistry in routine diagnostics. Thus, our findings may have immediate translational relevance for patients affected by this devastating disease.

## Supplementary Information

**Additional file 1**. Methods.

**Additional file 2: Supplementary Figure 1**. *RRM1, RRM2, *and* RRM2B *expression in EwS tumours, normal tissues and EwS cell line models, and their association with overall survival in 166 EwS patients.** Supplementary Figure 2**. RRM2 silencing inhibits cell proliferation and clonogenic growth in EwS in vitro, and representative immunohistochemical staining. **Supplementary Figure 3**. Development of chemoresistance in EwS cell lines, functional dependency of triapine on RRM2, and adverse effects of triapine treatment *in vivo*. **Supplementary Figure 4**. Drug interaction and combination efficiency of triapine with chemotherapeutics, PARP inhibitors, CHEK1 inhibitor or WEE1 inhibitor.

**Additional file 3: Supplementary Table 1**. Overexpressed genes in EwS compared to normal tissues and their prognostic relevance. **Supplementary Table 2**. Clinicopathological characteristics of EwS patients for the mRNA (discovery) and TMA-cohort (validation). **Supplementary Table 3**. Multivariate analysis for RRM2 expression and clinical parameters in the mRNA (discovery), TMA-cohort (validation) and joint-cohort. **Supplementary Table 4**. Commonly regulated genes upon *RRM2* silencing and pharmacological inhibition by triapine. **Supplementary Table 5**. Oligonucleotide sequences.

## Data Availability

Original microarray data used in this study were deposited at the National Centre for Biotechnology Information (NCBI) GEO under accession numbers GSE166415 and GSE166419. Custom code is available from the corresponding author upon reasonable request.

## Abbreviations

3-AP: 3-Aminopyridine-2-carboxaldehyde thiosemicarbazone
CC3: Cleaved caspase-3
CHEK1: Checkpoint kinase 1
CI: Combination index
Dox: Doxycycline
DR: Doxorubicin-resistant
EwS: Ewing sarcoma
EWSR1: Ewing sarcoma breakpoint region 1
FFPE: Formalin-fixed and paraffin-embedded
FLI1: Friend leukaemia virus integration 1
GO: Gene ontology
GR: Gemcitabine-resistant
GSEA: Gene set enrichment analysis
IHC: Immunohistochemistry
IRS: Immune reactive score
MSC: Mesenchymal stem cell
NSG: NOD/SCID/gamma
PARP: Poly ADP-ribose polymerase
RNR: Ribonucleotide reductase
RRM1: Ribonucleotide reductase catalytic subunit M1
RRM2: Ribonucleotide reductase regulatory subunit M2
RRM2B: Ribonucleotide reductase regulatory TP53 inducible subunit M2
TMA: Tissue microarray
TR: Triapine-resistant
WEE1: WEE1 G2 checkpoint kinase
WGCNA: Weighted gene co-expression network analysis
ZIP: Zero Interaction Potency
