# Supplementary material for: Translational evidence for RRM2 as a prognostic biomarker and therapeutic target in Ewing sarcoma
Source: Mol Cancer. 2021 Jul 27;20:97. doi: 10.1186/s12943-021-01393-9 (PMC8314608; doi:10.1186/s12943-021-01393-9)
Supplement: Supplementary file 1 — Additional file 1. Methods. [file 12943_2021_1393_MOESM1_ESM.docx]

**METHODS**

**Cell lines and cell culture conditions**

Human HEK293T cells and the human Ewing sarcoma (EwS) cell line A-673 were purchased from the American Type Culture Collection (ATCC). Human EwS cell lines SK-ES-1, and SK-N-MC, as well as the human osteosarcoma cell lines SaOS-2 and U2OS were provided by the German Collection of Microorganisms and Cell Cultures (DSMZ). Human EwS cell line TC-71 was kindly gifted by the Children’s Oncology Group (COG). Human EwS cell lines ES7 and EW-7 were provided by O. Delattre (Institute Curie, Paris). The human mesenchymal stem cell lines MSC-52 and MSC-72 originated from tumour-free bone marrow of EwS patients were provided by U. Dirksen (Essen, Germany). All cell lines except for MSC-52 and MSC-72 were maintained in RPMI-1640 media with stable glutamine (Biochrom, Germany) supplemented with 10% tetracycline-free fetal bovine serum (Sigma-Aldrich, Germany), 100 U/ml penicillin and 100 µg/ml streptomycin (Merck, Germany) in a humidified incubator with 5% CO_2_ at 37°C. MSC-52 and MSC-72 were maintained in Alpha MEM (Biochrom, Germany) supplemented with 10% tetracycline-free fetal bovine serum, 100 U/ml penicillin and 100 µg/ml streptomycin, 1% L-glutamine (Thermo Fisher Scientific) and 2 ng/ml recombinant human FGF-Basic (Thermo Fisher Scientific) in a humidified incubator with 5% CO_2_ at 37°C. All cell lines were routinely tested for the absence of mycoplasma contamination by nested-PCR. STR-profiling was regularly performed to assess cellular identity.

**Chemical compounds**

CCT245737, doxorubicin, etoposide, gemcitabine, MK-1775, niraparib, olaparib, triapine (3-AP), and vincristine were purchased from Selleckchem, resolved in DMSO and stored at –80°C.

**Assessment of mRNA expression with quantitative real-time polymerase chain reaction (qRT-PCR)**

Total RNA was isolated using NucleoSpin RNA kit according to the manufacturer’s instruction (Macherey-Nagel, Germany). One µg of total RNA was reverse-transcribed with High-Capacity cDNA Reverse Transcription Kit (Applied Biosystems, USA). cDNA was diluted at 1:10 in ddH_2_O and stored at -20°C. For qRT-PCR 6.75 µl cDNA was mixed with 7.5 µl SYBR green Master Mix (Applied Biosystems), 0.75 µl forward and reverse primer (10 µM) in a total volume of 15 µl. qRT-PCR reactions were performed with a BioRad CFX Connect instrument and the following thermal cycles; heat activation at 95°C for 2 min, denaturation at 95°C for 10 sec, annealing and elongation at 60°C for 20 sec (50 cycles), final denaturation at 95°C for 30 sec. Data was analysed by BioRad CFX Manager 3.1 software. For assessment of mRNA expression levels, the 2^−ΔΔCt^ method (1) was employed normalized to the housekeeping gene *RPLP0*. Oligonucleotides were purchased from MWG Eurofins Genomics (Ebersberg, Germany). Sequences are listed in **Supplementary Table 5**.

**Doxycycline (Dox)-inducible target knockdown by RNA interference with short hairpin RNA (shRNA)**

The Tet-pLKO-puro all-in-one vector (RRID: Addgene_21915) including a puromycin resistance cassette and a tet-responsive element for expression of shRNAs was established according to a publicly available protocol (2) using In-Fusion HD Cloning Kit (Clontech) (**Supplementary Table 5**). Vectors were amplified in Stellar Competent Cells (Clontech). Successful shRNA integration was verified by Sanger sequencing (primer: 5’-GGCAGGGATATTCACCATTATCGTTTCAGA-3’). Packaging plasmids pCMV-dR8.91 or psPAX2 (RRID: Addgene_12260), envelope plasmid pMD2.G (VSV-G) (RRID: Addgene_12259) and Tet-pLKO-puro all-in-one vectors harbouring shRNAs against *RRM2* (shRRM2) or a non-targeting control shRNA (shControl) were transfected in HEK293T. Lentiviral particles were yielded by filtering the supernatant and infected in EwS cell lines A-673, ES7, and TC-71, followed by resistant cell selection with 1 µg/ml puromycin (InVivoGen, USA). From puromycin-selected cells single cell clones were established and those with sufficient knockdown levels (approximately onto 30% remaining expression) were used for further analyses. Knockdown induction was achieved by adding 0.1 µg/ml Dox in cell culture media every 48h. Established cell lines were designated as cell line/TR/shRRM2_1, cell line/TR/shRRM2_3, cell line/TR/shControl.

**Proliferation assays**

2–3×10^4^ cells per well (depending on the cell line) were seeded on a 6-well plate in three technical replicates. 0.1 µg/ml Dox was added to media every 48h for knockdown induction. After harvesting cells including the supernatant, vital and dead cells were counted with a cell counter (Countess™ II Automated Cell Counter, Invitrogen) using Trypan-Blue exclusion method (Sigma-Aldrich).

**Clonogenic growth assays**

Cells were seeded at a density of 0.5–5×10^3^ per well (depending on the cell line) on a 6-well plate in three technical replicates and grown for 9–12 days. For genetic target inhibition 0.1 µg/ml Dox was added to cell culture media every 48h. For pharmacological target inhibition cells were treated with either drugs in indicated doses or equimolar vehicle (DMSO). Cell culture media were changed and drugs were renewed every 72h. Colonies were stained with crystal violet solution (Sigma-Aldrich). The colony area and intensity were calculated with the ImageJ Plugin *Colony area* (3).

**Establishment of drug-resistant EwS cells**

Doxorubicin-, gemcitabine-, and triapine-resistant EwS cells (DR, GR, TR, respectively) were established by culturing parental cells with serially increased concentration of each drug. Parental cells were treated with each drug from initial concentrations equivalent to IC_50_ values as assessed by Resazurin cell viability assays. Cells were deemed to have successfully adapted to the increment once they started to constantly regrow. The degree of drug resistance was assessed by IC_50_ values as determined by Resazurin cell viability assays.

**Transcriptome profiling, gene set enrichment analysis (GSEA), and gene ontology (GO) analysis**

For transcriptome profiling experiments, 1.0–2.0×10^5^ A-673/TR/shRRM2_1, A-673/TR/shRRM2_3, A‑673/TR/shControl, ES7/TR/shRRM2_1, ES7/TR/shRRM2_3 and ES7/TR/shControl were seeded in T-25 flasks, and treated with 0.1 µg/ml Dox for 72h (A-673) and 96h (ES7). For pharmacological RRM2 inhibition A-673 and ES7 were treated with either triapine (IC_50_ for A-673 0.44 µM, for ES7 0.65 µM) or equimolar vehicle (DMSO) for 72h. Total RNA was extracted using the NucleoSpin RNA kit according to the manufacturer’s instruction (Macherey-Nagel, Germany). Samples with RNA integrity numbers (RIN) >9 were hybridized to Human Affymetrix Clariom D microarrays at IMGM Laboratories (Munich, Germany). Data were quantile-normalized with Transcriptome Analysis Console (v4.0; Thermo Fisher Scientific) using the SST-RMA algorithm as previously described (4). For gene annotation the Affymetrix library for Clariom D Array (version 2, human) was employed.

For identification of differentially expressed genes (DEGs) with consistent and significant fold changes (FCs) across shRNAs and cell lines, genes with log2 transformed gene expression values lower than that of *ERG* (mean log2 expression around 6.0), which is virtually not expressed in *EWSR1-FLI1* positive EwS cell lines (5) were excluded. The FCs in shControl or vehicle, and two specific shRNAs or triapine treatment samples were individually calculated for each cell line. The FCs in shControl or vehicle samples were subtracted from those of shRRM2 or triapine treatment samples, respectively, yielding the cell line specific FCs for two specific shRNAs or triapine treatment. To integrate FCs across shRNAs or cell lines average FCs were used for further analyses. For downstream analyses, those genes with a minimum absolute log2 FC of 0.5 were included. To identify enriched gene sets, genes were ranked by the FC values, and a pre-ranked GSEA (MSigDB v7.0, c2.cpg.all) with 1,000 permutations was performed. To analyse enriched gene sets and their correlation upon RRM2 silencing, Weighted Gene Correlation Network Analysis (WGCNA) was performed (6) using Gene Ontology (GO) biological processes terms from MSigDB (c5.all.v7.0.symbols.gmt). Enriched GO terms were filtered for statistical significance (adjusted *P*<0.05) and a normalized enrichment score |(NES)|>1.5 (10,000 permutations). The constructed correlation network was visualized using Cytoscape (7).

For GO enrichment analysis under the condition of *RRM2* silencing or pharmacological inhibition in each cell line, commonly regulated genes in both cell lines and two experimental settings were extracted using Draw Venn Diagram (Van de Peer Labhttp://bioinformatics.psb.ugent.be/webtools/Venn/. Accessed 25 September 2020) (8). The commonly regulated genes were then interrogated for overrepresentation of biological processes using GO enrichment analysis (9-11). For gene co-expression analysis, the gene expression correlation between *RRM2* and other genes from 166 EwS tumours was estimated by calculating Pearson correlation coefficients. Those genes with |r_Pearson_| > 0.5 were further subjected to GO enrichment analysis. Heat maps were created by Morpheus (https://software.broadinstitute.org/Morpheus. Accessed 20 January 2021) (12) using the Pearson correlation coefficients between *RRM2* and other genes. Gene expression data were deposited at the GEO (accession codes: GSE166415 and GSE166419).

**Murine xenograft model**

2–2.5×10^6^ cells resuspended in a mix of 1:1 PBS and Geltrex Basement Membrane Mix (Thermo Fisher Scientific) were subcutaneously injected in the right flank of 10–12 weeks old NOD/Scid/gamma (NSG) mice. Tumour growth was assessed by measuring tumour size in two-dimension with a caliper every 2–3 day. The tumour volume was estimated by the formula length×(width)^2^/2. Once subcutaneous tumours became palpable (approx. 5 mm in diameter), animals were randomized in treatment group or control group. For induction of the *RRM2* knockdown 2 mg/ml Dox (BelaDox, Bela-pharm, Germany) was added in drinking water containing 5% sucrose (Sigma-Aldrich) for *ad libitum* uptake (Dox (+)), whereas the control group received sucrose alone (Dox (–)). For pharmacological RRM2 inhibition the treatment group received intraperitoneal injection of triapine 30 mg/kg every second day after the initial injection on two consecutive days, while the control group received vehicle (DMSO) alone. The endpoint was set at reaching an average tumour diameter of 1.5 cm, where animals were sacrificed by cervical dislocation. Any animals were excluded in case no subcutaneous tumour growth was observed.

**Cell viability assays and dose response assessment**

1.5–5×10^3^ cells per well (depending on the cell line) were seeded on a 96-well plate and treated with compounds in a dose range for which clinically achievable doses were taken into account if applicable. Assays were performed in a total volume of 100 µl on at least three technical replicates. Cell viability was assessed 84h after the treatment start with Resazurin cell viability assays (16 µg/ml, Sigma-Aldrich). Dose response curves were simulated by nonlinear regression models and IC_50_ values were calculated using PRISM 9 (GraphPad Software Inc., CA, USA) by normalizing to the respective controls (vehicle alone).

**Assessment of drug interaction and combination efficiency**

1.5–5×10^3^ cells per well (depending on the cell line) were seeded on a 96-well plate in triplicate and treated with various drug combinations either in constant dose ratios or in three serial doses (4×4 matrices). Cell viability was assessed 84h after the treatment start with Resazurin cell viability assays (16 µg/ml, Sigma-Aldrich) normalized to the respective controls (vehicle alone). The combination efficiency was analysed using the Chou-Talalay method (13) with CompuSyn, in which the combination index (CI) was calculated based on the dose-effect property for single drugs and their combinations. Dose-effect curves were linearized with the median-effect-plot for each single drug and combinations, and doses which cause the equivalent effect (reduction of cell viability) by the single drugs or combinations were calculated. CI is given by the formula: CI= (D)_1_/(Dx)_1_+(D)_2_/(Dx)_2_. (Dx)_1_, doses of drug 1 alone which shows _X_% inhibition; (Dx)_2_, doses of drug 2 alone which shows _X_% inhibition; (D)_1_, dose portion of drug 1 in combination with drug 2, which shows _X_% inhibition; (D)_2_, dose portion of drug 2 in combination with drug 1, which shows _X_% inhibition. The combination efficiency was interpreted as: CI value < 1 indicative of synergistic, CI = 1 additive, and CI > 1 antagonistic. The estimated drug combination efficiency was calculated by SynergyFinder 2.0 (14) based on ZIP reference model. ZIP synergy score > 10, likely to be synergistic; between –10 and 10, likely to be additive; < –10, likely to be antagonistic.

**Histology, immunohistochemistry (IHC), and evaluation of immunoreactivity**

Formalin-fixed and paraffin-embedded (FFPE) tissue sections were routinely processed and stained with haematoxylin and eosin for histological assessment including tissue structure, cellular morphology, mitosis, and cell death. For the detection of γH2A.X (phosphor-S139) 4 μm FFPE tissue sections were cut followed by an antigen retrieval by heat treatment with Target Retrieval Solution (S1699, Agilent Technologies, Germany). Slides were incubated with a monoclonal anti-γH2A.X (phosphor-S139) primary antibody (rabbit, 1:8,000, ab81299, Abcam, UK) for 60 min at room temperature, followed by incubation with a monoclonal secondary horseradish peroxidase (HRP)-coupled horse-anti-rabbit antibody (ImmPRESS Reagent Kit, MP-7401, Vector Laboratories, Germany) with AEC+ (K346, Agilent Technologies, USA) as chromogen, counterstained by haematoxylin Gill’s formula (H-3401, Vector Laboratories, Germany). For detection of cleaved caspase 3, antigen retrieval was carried out by heat treatment with Target Retrieval Solution Citrate pH6 (S2369, Agilent Technologies). Slides were incubated with a polyclonal cleaved caspase 3 primary antibody (rabbit, 1:100; 9661, Cell Signaling, Frankfurt am Main, Germany) for 60 min at room temperature followed by incubation with a monoclonal secondary horseradish peroxidase (HRP)-coupled horse-anti-rabbit antibody with AEC+ as chromogen, counterstained by haematoxylin Gill’s formula. For detection of RRM2, antigen retrieval was performed by heat treatment with ProTaqs IV Antigen-Enhancer (Quartett, 401602392). Slides were incubated with a polyclonal anti-RRM2 primary antibody (rabbit, 1:500, atlas antibodies, HPA056994) for 60 min at room temperature followed by incubation with a monoclonal secondary horseradish peroxidase (HRP)-coupled horse-anti-rabbit antibody with AEC+ as chromogen, counterstained by haematoxylin Gill’s formula (H-3401, Vector Laboratories, Germany). Slides were scanned using a Nanozoomer-SQ Digital Slide Scanner (Hamamatsu Photonics K.K.) and visualized with NDP.view2 image viewing software (Hamamatsu Photonics K.K.).

For assessment of RRM2 expression levels, immunoreactivity was semi-quantified as previously described in analogy to the hormone receptor scoring system Immune Reactive Score (IRS) with slight modifications to account for a 5-tier grading of positive tumour area (15, 16). First, the percentage of immunoreactive tumour cells was graded as follows; grade 0 = 0−19%, grade 1 = 20−39%, grade 2 = 40−59%, grade 3 = 60−79% and grade 4 = 80−100%. Second, the relative intensity of immunoreactivity was classified as follows; grade 0 = none, grade 1 = low, grade 2 = moderate and grade 3 = strong. IRS was then given as the product of both grades. For assessment of cleaved caspase-3 (CC3) and γH2A.X, immunoreactive cells were counted under the microscope in ten high power fields.

**Statistical analyses**

Statistical data analyses were performed using PRISM 9 (GraphPad Software Inc., CA, USA) on the raw data. For hypothesis tests for two groups a two-sided Mann-Whitney test was used if not otherwise specified in the figure legends. A Fisher exact probability test was employed for contingency data sets. To analyse bivariate correlations, Pearson correlation coefficients were calculated and analysed with PRISM 9. Data are presented as scatter-bar-plots with horizontal bars indicating means, and whiskers indicating the standard error of the mean (SEM), if not otherwise specified in the figure legends. The sample size for *in vitro* experiments was chosen empirically. The sample size for *in vivo* experiments was predetermined using power calculations with *β* = 0.8 and *α* <0.05 based on preliminary data and in compliance with the 3R system (replacement, reduction, refinement). For survival analysis, overall survival for clinical data sets or event-free survival for *in vivo* experiments were described by Kaplan-Meier curves, and survival functions between the groups were analysed by a log-rank test or a Mantel-Haenszel test. *P*-values < 0.05 were considered as statistically significant. *P*-values were calculated from two-sided statistical tests, if not otherwise specified in the figure legends.

**REFERENCES**

1. Livak KJ, Schmittgen TD. Analysis of relative gene expression data using real-time quantitative PCR and the 2(-Delta Delta C(T)) Method. Methods. 2001;25(4):402-8.

2. Wiederschain D, Wee S, Chen L, Loo A, Yang G, Huang A, et al. Single-vector inducible lentiviral RNAi system for oncology target validation. Cell Cycle. 2009;8(3):498-504.

3. Guzmán C, Bagga M, Kaur A, Westermarck J, Abankwa D. ColonyArea: an ImageJ plugin to automatically quantify colony formation in clonogenic assays. PLoS One. 2014;9(3):e92444.

4. Machiela MJ, Grünewald TGP, Surdez D, Reynaud S, Mirabeau O, Karlins E, et al. Genome-wide association study identifies multiple new loci associated with Ewing sarcoma susceptibility. Nat Commun. 2018;9(1):3184.

5. Crompton BD, Stewart C, Taylor-Weiner A, Alexe G, Kurek KC, Calicchio ML, et al. The genomic landscape of pediatric Ewing sarcoma. Cancer Discov. 2014;4(11):1326-41.

6. Langfelder P, Horvath S. WGCNA: an R package for weighted correlation network analysis. BMC Bioinformatics. 2008;9:559.

7. Shannon P, Markiel A, Ozier O, Baliga NS, Wang JT, Ramage D, et al. Cytoscape: a software environment for integrated models of biomolecular interaction networks. Genome Res. 2003;13(11):2498-504.

8. Lab VdP. Draw Venn Diagram Available from: http://bioinformatics.psb.ugent.be/webtools/Venn/.

9. Mi H, Muruganujan A, Ebert D, Huang X, Thomas PD. PANTHER version 14: more genomes, a new PANTHER GO-slim and improvements in enrichment analysis tools. Nucleic Acids Res. 2019;47(D1):D419-d26.

10. Ashburner M, Ball CA, Blake JA, Botstein D, Butler H, Cherry JM, et al. Gene ontology: tool for the unification of biology. The Gene Ontology Consortium. Nat Genet. 2000;25(1):25-9.

11. The Gene Ontology resource: enriching a GOld mine. Nucleic Acids Res. 2021;49(D1):D325-d34.

12. Institute B. Morpheus Available from: https://software.broadinstitute.org/morpheus.

13. Chou TC, Talalay P. Quantitative analysis of dose-effect relationships: the combined effects of multiple drugs or enzyme inhibitors. Adv Enzyme Regul. 1984;22:27-55.

14. Ianevski A, Giri AK, Aittokallio T. SynergyFinder 2.0: visual analytics of multi-drug combination synergies. Nucleic Acids Res. 2020;48(W1):W488-w93.

15. Marchetto A, Ohmura S, Orth MF, Knott MML, Colombo MV, Arrigoni C, et al. Oncogenic hijacking of a developmental transcription factor evokes vulnerability toward oxidative stress in Ewing sarcoma. Nat Commun. 2020;11(1):2423.

16. Baldauf MC, Orth MF, Dallmayer M, Marchetto A, Gerke JS, Rubio RA, et al. Robust diagnosis of Ewing sarcoma by immunohistochemical detection of super-enhancer-driven EWSR1-ETS targets. Oncotarget. 2018;9(2):1587-601.
