# Supplementary material for: Translational evidence for RRM2 as a prognostic biomarker and therapeutic target in Ewing sarcoma
Source: Mol Cancer. 2021 Jul 27;20:97. doi: 10.1186/s12943-021-01393-9 (PMC8314608; doi:10.1186/s12943-021-01393-9)

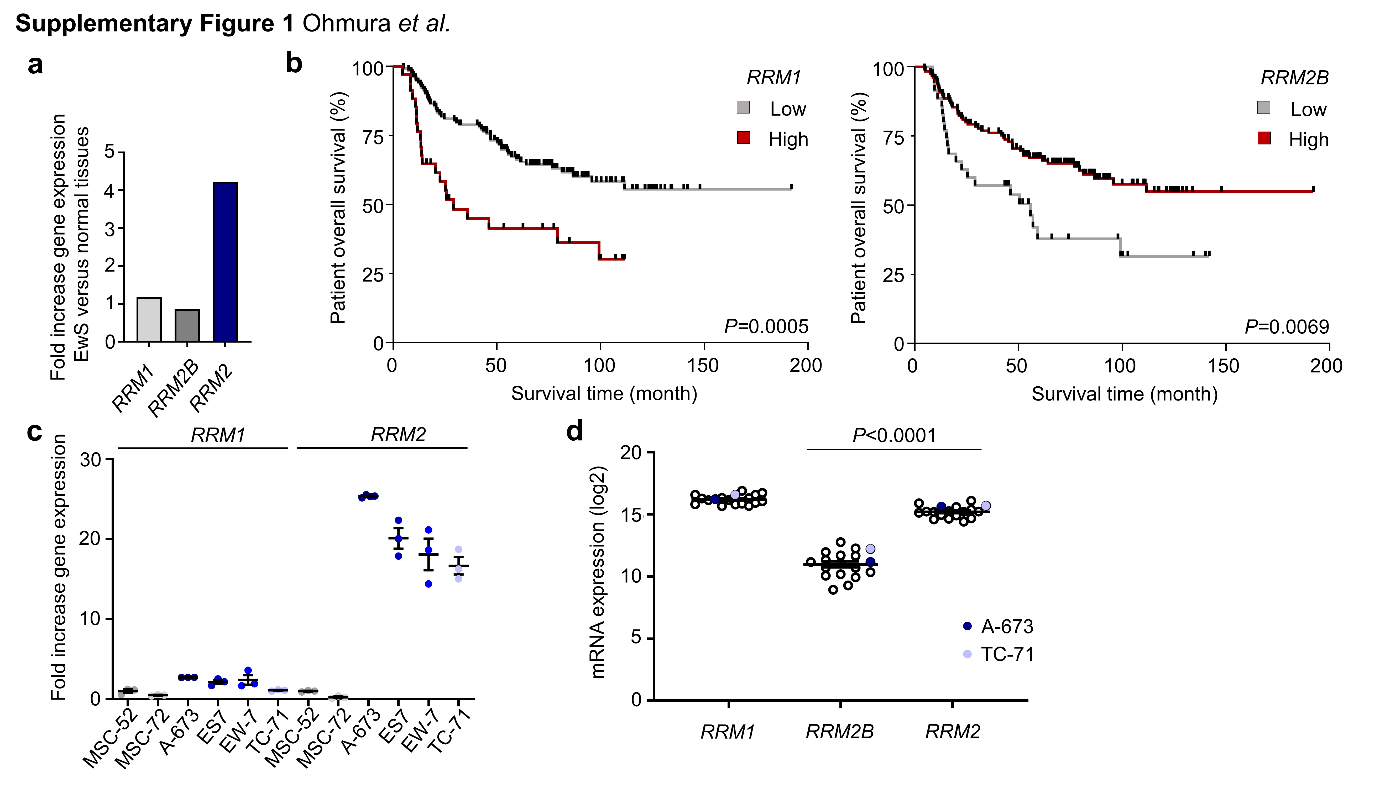


**Supplementary Figure 1 | *RRM1, RRM2,* and *RRM2B* expression in EwS tumours, normal tissues, and EwS cell line models, and their association with overall survival in 166 EwS patients.**

**a)** Analysis of *RRM1*, *RRM2,* and *RRM2B* mRNA expression levels in 50 EwS primary tumours compared to 929 normal tissues samples from 71 tissue types. Data are shown by fold increase normalized to expression values of normal tissues. **b)** Analysis of overall survival time of 166 EwS patients for *RRM1* and *RRM2B* mRNA expression. *P*-values were determined in Kaplan-Meier analyses using a Mantel-Haenszel test. **c)** Analysis of *RRM1* and *RRM2* mRNA expression levels in EwS cell lines (A-673, ES7, EW-7, TC-71) compared to control cells (mesenchymal stem cells, MSC-52, MSC-72). Data are shown by fold increase normalized to expression values of MSC-52. **d)** Analysis of *RRM1*, *RRM2,* and *RRM2B* mRNA expression levels in 18 EwS cell lines obtained from publicly available transcriptome data (Orth *et al.* 2021 bioRxiv <https://doi.org/10.1101/2021.06.08.447518>; GSE176339) including A-673 and TC-71.


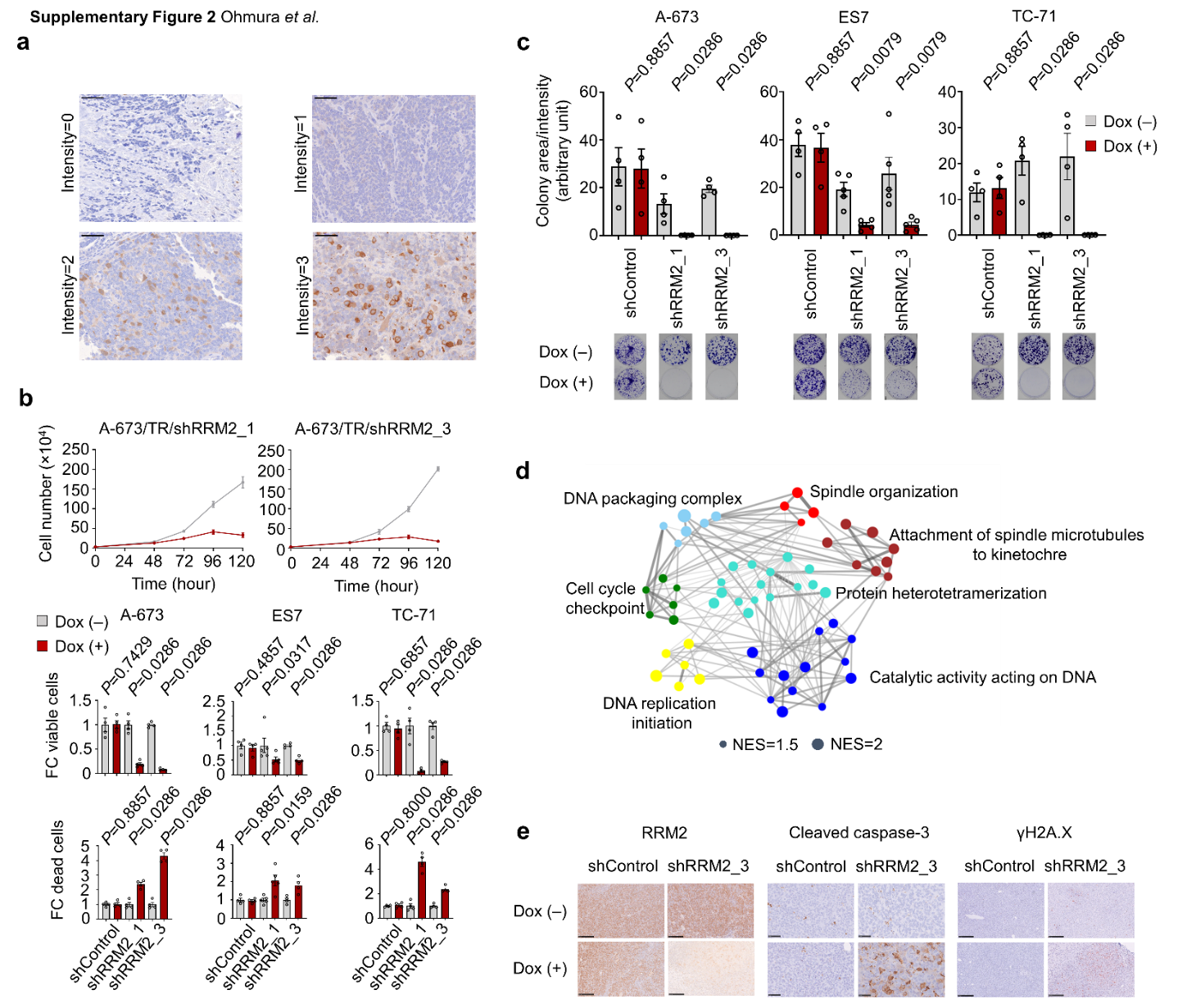


**Supplementary Figure 2 | *RRM2* silencing inhibits cell proliferation and clonogenic growth in EwS *in vitro*, and induces apoptosis and DNA damage *in vivo*.**

**a)** Representative micrographs of immunohistochemical RRM2 staining for tissue micro arrays. Scale bar = 50 µm. **b)** Analysis of proliferation assays upon shRNA-mediated *RRM2* silencing in EwS cell lines. Upper: Cell proliferation over 120h upon *RRM2* silencing in A-673. Viable cells upon *RRM2* silencing (middle) and dead cells (lower) in EwS cell lines (A-673, ES7, TC-71) harbouring Dox-inducible shRRM2 constructs or non-targeting shRNA (shControl). Values were normalized to Dox (–). Horizontal bars represent means and whiskers SEM. FC, fold change. Two-sided Mann-Whitney test. **c)** Analysis of clonogenic growth upon shRNA-mediated *RRM2* silencing in EwS cell lines (A-673, ES7, TC-71) harbouring Dox-inducible shRRM2 constructs or non-targeting shRNA (shControl). Horizontal bars represent means and whiskers SEM. Two-sided Mann-Whitney test at the experimental endpoint. **d)** WGCNA of downregulated genes upon *RRM2* silencing in A-673 and ES-7 cells harbouring Dox-inducible shRRM2 constructs. NES, normalized enrichment score. **e)** Representative micrographs of xenografts immunohistochemically stained for RRM2, cleaved caspase-3 (CC3) or γH2A.X (scale bar=250 µm, 50 µm, 250 µm, respectively).


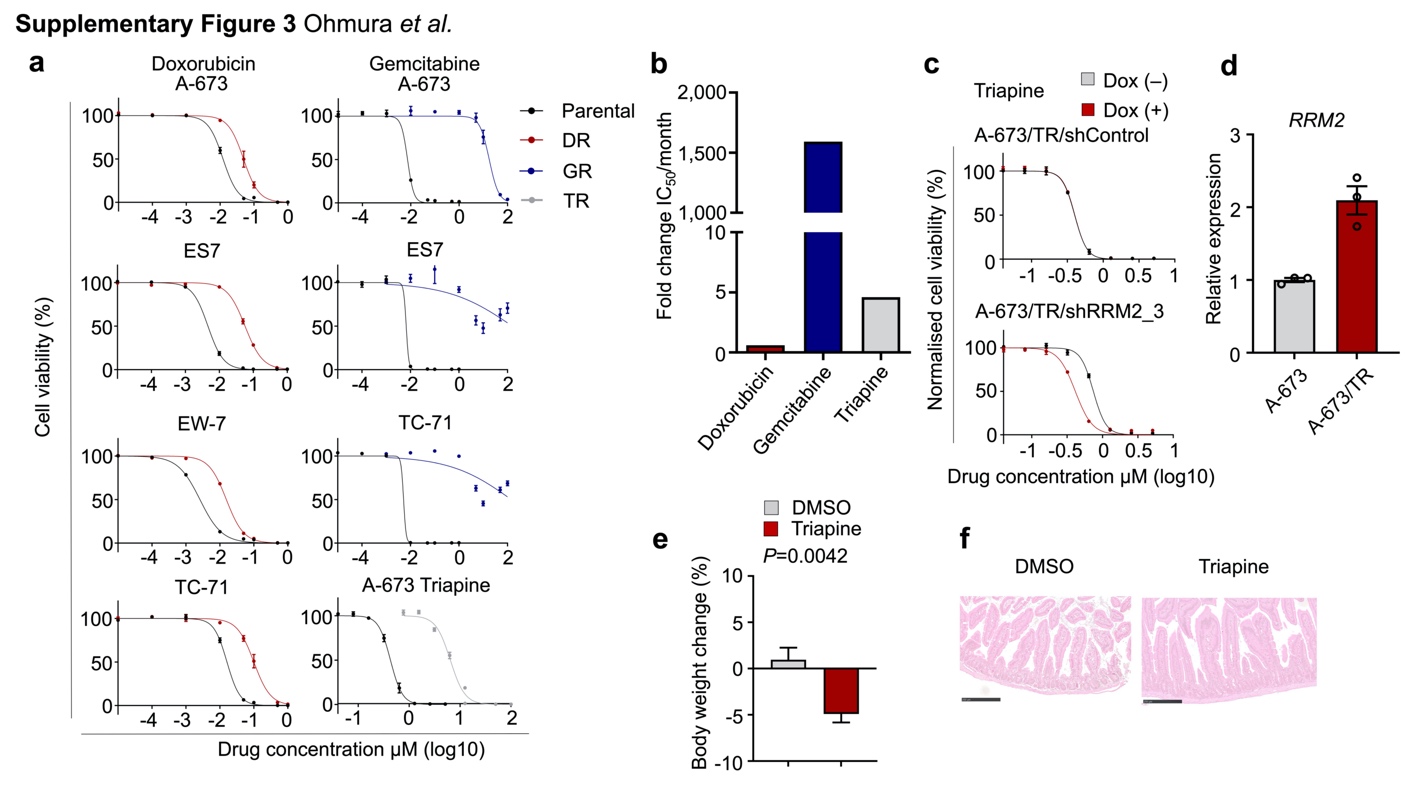


**Supplementary Figure 3 | Development of chemoresistance in EwS cell lines, functional dependency of triapine on RRM2, and adverse effects of triapine treatment *in vivo*.**

The EwS cell lines were subjected to serially ascending doses of either doxorubicin (A-673, ES7, EW-7, TC-71), gemcitabine (A-673, ES7, TC-71) or triapine (A-673). **a)** Comparative drug-response analysis for doxorubicin, gemcitabine or triapine in parental, doxorubicin-resistant (DR), gemcitabine-resistant (GR) or triapine-resistant (TR) cells assessed by Resazurin cell viability assays. **b)** Drug-resistance developing rate for doxorubicin, gemcitabine or triapine in A-673. Data are shown by fold change in IC_50_ normalized to those of the parental cells divided by time for stably acquiring drug resistance. **c)** Dose response analysis for triapine upon *RRM2* silencing or induction of shControl in A-673. For each condition data were normalized to vehicle (DMSO). **d)** *RRM2* expression in triapine-resistant A-673 (A-673/TR) compared to parental A-673 cells. **e)** Change of body weight upon triapine or vehicle treatment at the experimental endpoint. Data are shown by body weight change before and after treatment normalized to the baseline (initial values before starting treatment). **f)** Histological assessment of adverse effects in the intestine. Haematoxylin and Eosin staining. Scale bar=250 µm.

**Supplementary Figure 4 | Drug interaction and combination efficiency of triapine with chemotherapeutics, PARP inhibitors, CHEK1 or WEE1 inhibitor.**

**a)** Drug interaction and combination efficiency analysis between triapine and doxorubicin, etoposide, vincristine, olaparib or niraparib in four EwS cell lines (A-673, ES7, EW-7, TC-71) assessed by combination index. CI value < 1 indicative of synergistic, CI = 1 additive, and CI > 1 antagonistic. **b)** Drug interaction and combination efficiency estimation between triapine and CHEK1 inhibitor (CCT245737) or WEE1 inhibitor (MK-1775) in A-673 EwS cell line assessed by SynergyFinder 2.0. Upper: Dose response matrix. Lower: Synergy distribution. ZIP synergy score > 10, likely to be synergistic; between –10 and 10, likely to be additive; < –10, likely to be antagonistic.
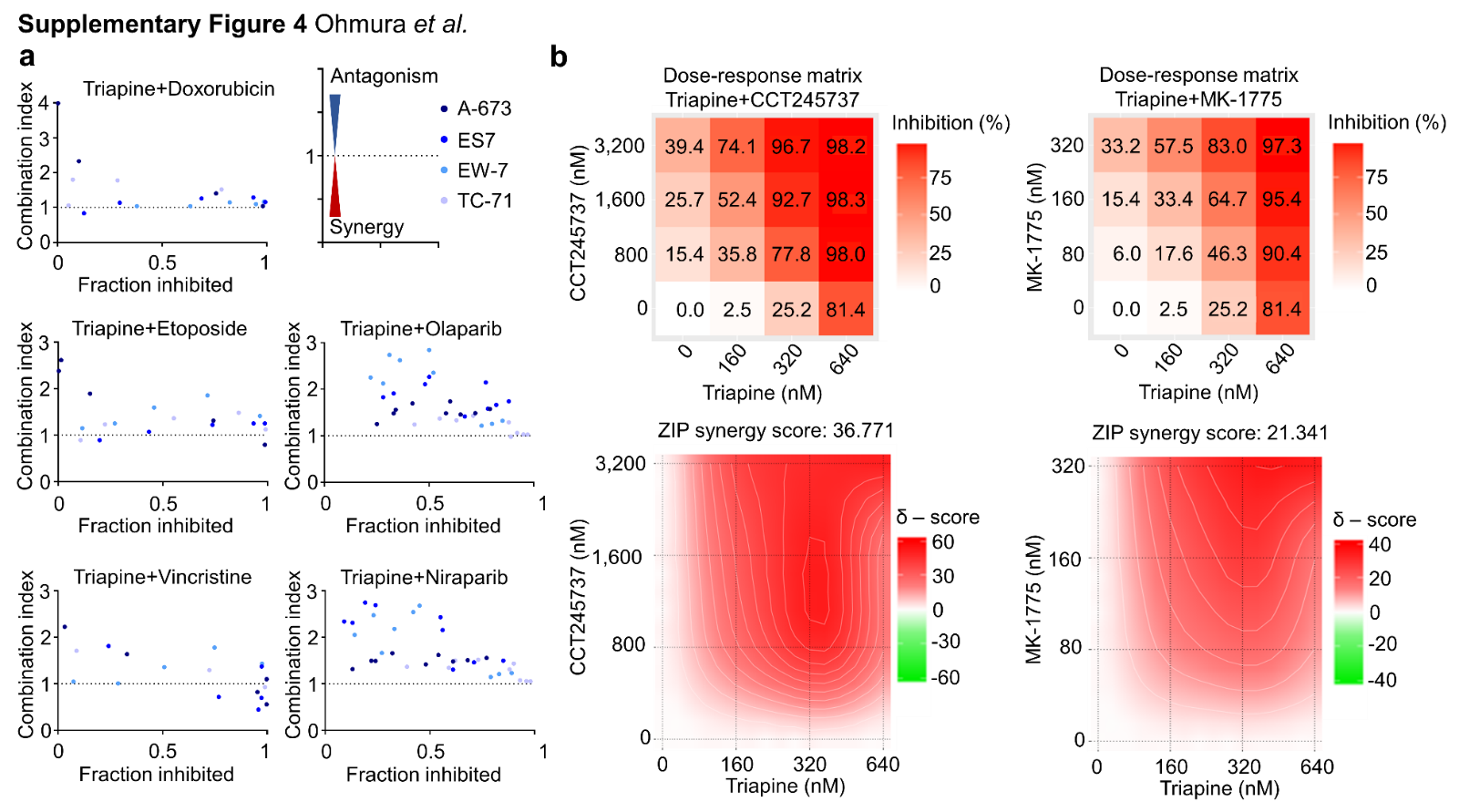

Supplement: Supplementary file 2 — Additional file 2: Supplementary Figure 1. RRM1, RRM2, and RRM2B expression in EwS tumours, normal tissues and EwS cell line models, and their association with overall survival in 166 EwS patients. Supplementary Figure 2. RRM2 silencing inhibits cell proliferation and clonogenic growth in EwS in vitro, and representative immunohistochemical staining. Supplementary Figure 3. Development of chemoresistance in EwS cell lines, functional dependency of triapine on RRM2, and adverse effects of triapine treatment in vivo. Supplementary Figure 4. Drug interaction and combination efficiency of triapine with chemotherapeutics, PARP inhibitors, CHEK1 inhibitor or WEE1 inhibitor. [file 12943_2021_1393_MOESM2_ESM.docx]
